# The I2 Statistic As Selection Bias Test: Updated Threshold Limits

**DOI:** 10.7759/cureus.107553

**Published:** 2026-04-22

**Authors:** Steffen Mickenautsch, Veerasamy Yengopal

**Affiliations:** 1 Faculty of Dentistry, University of the Western Cape, Cape Town, ZAF; 2 Community Dentistry, University of the Witwatersrand, Johannesburg, ZAF

**Keywords:** bias test, i2 test, randomized control trials, review of clinical trials, selection bias, systematic review and meta analysis

## Abstract

Aim: This study aims to revise the pre-specified I^2^ point estimate threshold limits of the trial-adjusted, simulated comparator trial (SCT)-based I^2^ test for selection bias in single randomised controlled trials (RCTs), in order to increase the percentage of testable RCTs. A further objective was to test the two null hypotheses: that, based on the test’s revised I^2^ point estimate thresholds, the magnitude of trial effect estimates is not significantly positively correlated with the selection bias levels (H01), and that it does not differ significantly between RCTs with identified ‘low’ and ‘high’ selection bias (H02).

Methods: Revision of the I^2^ point estimate threshold limits was based on multiple RCT simulation and the re-testing of 332 real-world RCTs for selection bias. The selection bias level (B%) of each RCT, using the revised limits, was determined. H01 was tested using Spearman’s rank correlation, and H02 using the 2-tailed, independent samples t-test. The mean difference (MD) with 95% confidence interval (CI) between the mean absolute risk difference (RD) values with SD of RCTs with identified ‘low’ and ‘high’ selection bias was computed.

Results: All 332 RCTs were testable, and none of the computed I^2^ point estimates fell outside the revised thresholds limits, increasing the proportion of testable RCTs from 71% to 100%. There was a statistically significant, positive, small correlation (0.1 ≤ |r| < 0.3) between the absolute RD values and established B% levels (Spearman’s rho = 0.268, p < 0.0001). The mean absolute RD for RCTs with identified ‘high’ selection bias was 0.18 (SD = 0.16), compared with 0.10 (SD = 0.13) for RCTs with ‘low’ selection bias. The difference was statistically significant: (t = -4.65; p < 0.0001; MD 0.08; 95%CI: 0.05 - 0.11). The effect size (Cohen's d = 0.52) indicates a medium effect. Both null hypotheses were rejected.

Conclusion: The revised threshold limits increased the utility of the trial-adjusted, SCT based I^2^ test from 71 to 100% testable RCTs. The rejection of both null hypotheses indicates that the previously established statistically significant, positive relationship between selection bias levels (B%) and effect estimates (absolute RD) is independent of the changes in the I² threshold limits.

## Introduction

Selection bias in randomised controlled trials (RCTs) represents one type of systematic error that is generated by either the non-random, erroneous or willful allocation of patients with known treatment-success supporting characteristics to one of the trial treatment groups. This can lead to a more favourable outcome in one group above compared to the other, independent of the true clinical effect of the treatments being evaluated [1].

Mickenautsch and Yengopal developed a test for detecting selection bias in single RCTs [2]. The test relies on data from baseline variables of the compared treatment groups and uses the I^2^ point estimate to signal whether selection bias risk is present (I^2^ > 0%), as well as to estimate the percentage of trial subjects that have been non-randomly allocated for the benefit of one treatment group above the other. The rationale of the test is based on the premise that effective randomisation in RCTs assures a lack of in-between study heterogeneity beyond the play of chance, which is reflected as I^2^ = 0% in a baseline variable meta-analysis [3,4].

Clark et al. established that an increased I^2^ point estimate above 0% in a baseline variable meta-analysis indicates imbalances in baseline variable values between treatment groups in an RCT. They noted that, unlike in an outcomes meta-analysis, such an increase could only be attributed to the presence of selection bias [3]. Accordingly, Hicks et al. developed a selection bias test for outcome meta-analyses. This method involves including baseline measurements from the treatment groups of several RCTs in a fixed effect baseline variable meta-analysis of continuous data, computing the t-statistic for each RCT, and then stepwise removing RCTs with the largest t-statistic from the meta-analysis until I^2^ = 0% [5].

Mickenautsch and Yengopal proposed that in place of several RCTs, two bias-free simulated comparator trials (SCTs) could be generated and included together with baseline data of a single RCT into a baseline variable meta-analysis, instead. In that way the testing of single RCTs for selection bias becomes possible [2]. In addition, it was found that modelling the included SCT data in accordance to the baseline data of the tested RCT (i.e.: ‘trial-adjusted’) increased the odds for selection bias detection [6]. In addition to merely identifying selection bias in RCTs, it was further observed that changes in the trial sample size (n_i_) uniquely affect the I^2^ point estimate value under condition of different simulated selection bias levels (B%). These levels can, therefore, be identified from I^2^ readings within certain pre-specified I^2^ threshold limits [7]. The simulated selection bias levels (B%) represent the percentages of trial subjects that are non-randomly allocated in support of one intervention group above the other. A simulation study found that the percentage of RCTs with statistically significant effect estimates increased from 0% at B% 0-30 to 100% at B% 40-100 [8]. Accordingly, the practical distinction between ‘low’ (B% 0- 30) versus ‘high’ (B% 40-100) selection bias can empirically be justified.

The trial-adjusted, SCT based I^2^ test developed from these findings proved be a more effective method for detecting selection bias in RCTs than common p-value-based significance testing, or the use of Cochrane’s Risk of Bias tool (RoB 2) [8,9]. However, when used on 332 real-world RCTs, only 235 of these trials could be tested within pre-specified I^2^ threshold limits [10]. The original thresholds consisted of a minimum/maximum ranges of I^2^ point estimates for three sample sizes, n_i_, that were specific for 11 selection bias levels (B% 0-100).Testing produced I² values that fell outside these limits for 97 RCTs, thereby preventing accurate bias classification in 29% of trials and limiting the utility of the test. For these reasons, more applicable I² threshold limits were required.

Accordingly, the aim of this study was to revise the test’s pre-specified I^2^ point estimate threshold limits in order to increase the proportion of RCTs that can be evaluated using threshold-guided selection bias level (B%) estimation. A further objective was to test the two null hypotheses: that, based on the test’s revised I^2^ point estimate thresholds, the magnitude of trial effect estimates is not significantly positively correlated with the selection bias levels (H01), and that it does not differ significantly between RCTs with identified ‘low’ and ‘high’ selection bias (H02).

This manuscript has been published as a preprint in Authorea [11].

## Materials and methods

Revision of the test’s I^2^ threshold limits

To revise the pre-specified I^2^ point estimate threshold limits of the trial-adjusted, SCT based I^2^ test, a total of 200 RCTs were simulated in MS Excel (Appendix 1, Section 1) [2,10]. Each simulated trial comprised three columns: (1) an ascending sequence of numbers from 1 to 200, representing trial subject IDs; (2) a random allocation sequence for two intervention groups (Group A and B), generated using the online tool ‘Sealed envelope’ with block randomisation, (block size = 4); (3) a sequence of random values generated using an online randomisation tool, with the following settings: Allow duplication in results = Yes; Total number of values = 200; Value type = Numbers with two decimals [12,13]. To ensure that the simulated random values were as realistic as possible, the lower and upper limits for value generation were defined based on baseline variable ranges observed in 332 real-world RCTs included in a previous study [10].

For this purpose, baseline values were extracted from the 332 trials and the minimum, the median and the maximum values of their SDs determined. For each of these values, the range of one SD was calculated by adding and subtracting the SD from the sample mean and the resulting minimum and maximum values used as lower and upper limit during random number generation for Column 3. In that way, 150 different sequences of ascending, random values were generated - 50 within the minimum, median and maximum baseline value ranges, each.

In the next step, the generated random allocation sequence, representing zero selection bias (B% 0), was artificially biased by sorting the first 20, 40, 60, 80, 100, 120, 140, 160, 180 and 200 of the total rows of column 2 in ascending order (from the lowest to the highest value), thus generating the bias levels B% 10, 20, 30, 40, 50, 60, 70, 80, 90 and 100, respectively. Because the values in Column 3 were already sorted in ascending order, this artificial bias in Column 2 (so that the sorted rows all indicated allocation to group ‘A’) ensured that lower values were allocated to group A than to B, thus causing an erroneously higher sample mean for group B. A total of 150 simulated RCTs were generated for each of the 11 bias levels (B% 0, 10, … 100) by assigning the 150 different sequences of ascending, random values to each (Appendix 1, Section 2).

For each simulated RCT, the random values from Column 3 were sorted according to the allocation sequences in Column 2, and the sample mean (with SD) was calculated for each group. Results were rounded to the second decimal. Following published I^2^ bias test methodology, two identical SCTs were generated in MS Excel from the described three columns at selection bias level zero (B% = 0) and their mean (SD) values for group A and B entered into a fixed-effect meta-analysis for continuous data [2]. The same type of data from the simulated RCT was added into the meta-analysis and the I^2^ point estimate computed for each of the sample size (n_i_) = 1, 2, 3 … to 100 per group for, both, SCTs and the simulated RCT, using Cochrane’s Review Manager (RevMan) software [14]. Applying this method to all 150 simulated RCTs in all 11 selection bias levels (B% 0-100) produced a total of 165,000 I^2^ data points, which were graphically presented in scatter plots for this study (Appendix 1, Section 3).

**Figure 1:**
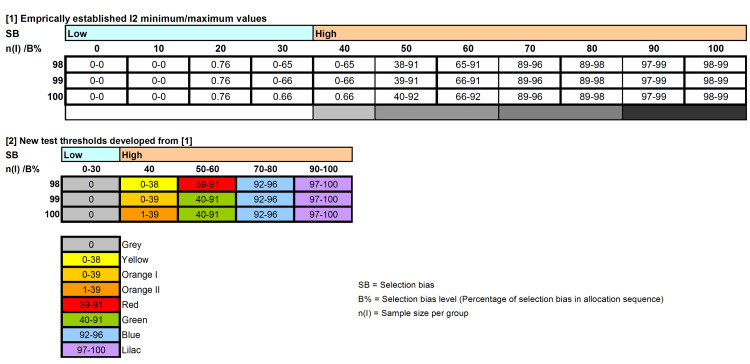
Established minimum/maximum I2 point estimates per bias level and new test thresholds Colors represent the specific minimum/maximum I^2^ point estimates per bias level that are displayed in each field. Image generated with MS PowerPoint and adjusted with Adobe Photoshop Elements 11.

All I^2^ data points were recorded in MS Excel, and the minimum-maximum values for each sample size (n_i_) and selection bias level (B%) were determined (Appendix 1, Section 3). Based on these data, and with a few justified deviations (Table 1), new I^2^ test threshold limits were developed for the sample sizes n_i_ = 98, 99 and 100 (Appendix 1, Section 4; Figure 1).

**Table 1:** Deviations of new test thresholds from established minimum/maximum I2 point estimates per bias level B% = Selection bias level; n_i_ = Sample size per treatment group

Deviation	Justification
1. Combining of bias levels B% 0, 10, 20 and 30	All four bias levels are associated with ‘low’ selection bias, without any significant effect on magnitude and direction of the outcome effect estimate All three generate zero I^2^ point estimates at n_i_ = 98, 99 and 100 in 98% of all simulated trials.
2. Combining of bias levels B% 50 and 60	Large overlaps of lower and upper threshold limits preventing any useful distinguish between the two bias levels on basis of the I^2^ point estimates.
3. Combining of bias levels B% 70 and 80	
4. Combining of bias levels B% 90 and 100	
5. Reduction of I^2^ > 0 point estimates in bias levels B% 20 and 30	I^2^ > 0% point estimates were only observed in 7 out of a total of 300 (2%) simulated trials in the two bias levels (all others: I^2^ = 0%), mainly due to rounding up of the 2nd digit after comma from the 3rd and 4th digit.
6. Minor increase of lower limit of combined bias level B% 40 at n_i_ = 100 from I^2^ = 0 to 1%	None, except the reduced point estimates (see point 2 above), reach I^2^ >0% at bias level B% 30, while 73% of all 150 simulated trial runs at bias level B% 40 do.
7. Reduction of upper limit of bias level B% 40 from I^2^ = 65 to 38, 39 and 39% at n_i_ = 98, 99 and 100, respectively	Although some overlap remains, only 23% of the 150 simulated trials at bias level B% 40 reach above the new upper limits, while one of all 150 simulated trials at bias level B% 50 reach below the new limits at n_i_ = 98 and 99, each and none at n_i_ = 100.
8. Minor reduction of upper limit of combined bias level B% 50-60 from I^2^ = 92 to 91% at n_i_ = 100	Although some overlap remains, only 6% of the 150 simulated trials at bias level B% 60 reach above, while a minority of 34% of all 150 simulated trials at bias level B% 70 reach below the reduced upper limit.
9. Minor reduction of upper limit of combined bias level B% 70-80 from I^2^ = 98 to 96% at n_i_ = 98, 99 and 100.	Although some overlap remains, only 6% of the 150 simulated trials at bias level B% 80 reach above, while none at bias level B% 90 reach below I^2^ = 97%.
10. Minor increase of upper limit of combined bias level B% 90-100 from I^2^ = 99 to 1100% at n_i_ = 98, 99 and 100.	The increase includes the maximal possible point estimate that can be reached.

These new limits differ from the original ones in that they use sample sizes (n_i_) 89, 99 and 100, instead of 10, 50 and 100, and that do not differentiate between lower bias levels (B% 0, 10, 20 and 30) [10]. Consequently, the new limits no longer include the highly inflated artificial sample sizes (n_i_) 5,000, 18,000 and 36,000 anymore. Furthermore, no distinction was made between the following bias levels: B% 50 versus 60, B% 70 versus 80 and B% 90 versus 100; these were, therefore, grouped into bias categories 3, 4 and 5 (with bias levels B% 0-30 and 40 as bias categories = 1 and 2, respectively (Appendix 1, Section 5)). 

Hypotheses testing

All 332 RCTs included in a previous study [10] were re-tested for selection bias using the trial adjusted, SCT-based I^2^ test [2] at sample sizes n_i_ = 98, 99 and 100. The resulting I^2^ point estimates were rated using the revised test thresholds (Figure 1).

As in the previous study [10], the relationship between the magnitude of the RCT effect estimates (absolute values of the risk difference (RD)) and the bias levels (B%) (H01) was assessed using Spearman’s rank correlation coefficient. For correlation testing, the new selection bias levels B% 0-30, 40, 50-60, 70-80 and 90-100 were converted into the bias categories with the values 1, 2, 3, 4 and 5, respectively. The bias category values formed the independent variable (x), and the absolute RD values from the RCTs formed the dependent variable (y) for analysis. 

The difference in absolute RD values between RCTs with ‘low’ (B% = 0-30) and ‘high’ (B% = 40-100) selection bias (H02) was evaluated using a 2-tailed independent samples t-test, with the alpha level was set at 5%. Analyses were performed in SAS (SAS Institute, USA). In addition, the mean difference (MD) with 95% confidence interval (CI) between the mean absolute RD values (with SD) from RCTs with identified ‘low’ and ‘high’ selection bias was calculated using Cochrane’s RevMan software [14].

## Results

All 332 RCTs could be tested and none of the computed I^2^ point estimates fell outside the revised, new test thresholds (Figure 1). Compared with the original test results, selection bias rating with the new threshold limits assigned 12 RCTs to a higher and seven of the 332 RCTs to a lower selection bias level (B%). Bias rating with the new test thresholds of all other 313 (94%) RCTs did not contradict the original test results of the previous study (Appendix 1, Section 5) [10].

**Figure 2:**
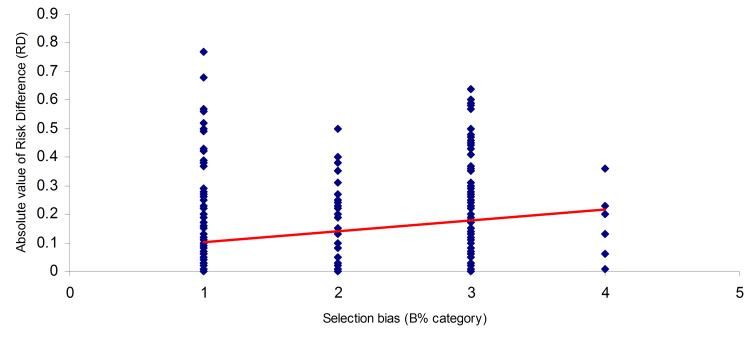
Scatterplot of correlation between trial effect estimates (absolute RD values) and selection bias B% category values Spearman’s rank correlation: r = 0.268, p < 0.0001 RD: Risk difference

There was a significant, positive, small (0.1 ≤ |r| < 0.3) correlation between the magnitudes of trial effect estimates (absolute RD values) and the selection bias B% category values: Spearman’s rho = 0.268, p < 0.0001 (Figure 2).

The magnitude of trial effect estimates, represented by the absolute mean RD, was 0.18 (SD = 0.16) for RCTs with identified ‘high’ selection bias (B% 40-100) and 0.10 (SD = 0.13) for RCTs with identified ‘low’ selection bias (B% 0-30). Effect estimates were significantly higher for RCTs with ‘high’ selection bias than for those with ‘low’ selection bias, with an MD of 0.08 (95% CI: 0.05-0.11; t = -4.65; p < 0.0001), corresponding to an eight-percentage-point increase. The effect size (Cohen's d = 0.52) indicates a medium effect (Appendix 1, Section 5). Both null hypotheses, H01 and H02, were rejected.

## Discussion

The aim of this study was to revise the pre-specified I^2^ point estimate threshold limits of the trial-adjusted, SCT-based I^2^ selection bias test, in order to increase the percentage of RCTs that are testable via threshold-guided selection bias level (B%) estimation [2,10]. A further objective was to test the two null hypotheses - that with the revised thresholds, the magnitude of trial effect estimates is not significantly positively correlated with the selection bias levels (H01), and that it does not differ significantly between RCTs with identified ‘low’ and ‘high’ selection bias (H02).

The results demonstrated that, compared with previous findings [10], all RCTs could be tested for selection bias within the new I^2^ threshold limits, thus increasing the percentage of testable trials from 71% to 100%. In addition, both null hypotheses were rejected. These findings suggest that revising the original thresholds increased the utility of the I^2^ statistic for selection bias testing of single RCTs, while confirming the original observations concerning the direct B% correlation with RD values (H01) and statistically significantly higher effect estimates for RCT with high selection bias (in comparison to low-bias RCTs). Previous study results showed a direct correlation between B% and RD for null hypothesis H01, with r = 0.304 (p < 0.0001) in the main analysis and r = 0.25 (p < 0.001) in the sensitivity analysis. For null hypothesis H02, there was a statistically significant MD in absolute RD between RCTs with ‘low’ and ‘high’ selection bias: MD = 0.094 (95% CI: 0.075-0.11) and MD = 0.07 (95% CI: 0.03-0.11) [10]. These results were based on I² threshold limits developed from a single RCT simulation, which did not account for variations in the values of baseline variables across multiple RCTs [7]. In the present study, I^2^ threshold limits were developed from multiple RCT simulations, including 50 different variable variations for three value domains - the minimum, maximum, and median values of baseline variables extracted from 332 real-world RCTs. Despite this higher precision in the thresholds, null-hypothesis testing yielded confirmatory results: H01: r = 0.268; p < 0.0001; H02: MD 0.08; 95% CI: 0.05-011. These findings indicate that the relationship between selection bias levels and effect estimates is independent of changes to the I^2^ threshold limits.

Importantly, the use of the new threshold limits eliminated the need to consider RCTs as untestable or to rely on ‘B% estimation by approximation’, a method in which the selection bias level (B%) must be guessed because not all established I² point estimates fall within the pre-specified thresholds for different B% levels. Such approximation was shown to produce erroneously lower B% values (MD -3.75; 95% CI: -3.75 to -0.03) and is therefore not recommended [10]. Furthermore, the new I^2^ threshold limits (Figure 1) accommodated most (94%) of the bias ratings from the old limits, while reclassifying 12 RCTs to higher and only seven RCTs to lower bias levels. This may suggest that the new I^2^ threshold limits are somewhat more stringent. These results also indicate that the findings of this study do not invalidate the test outcomes of RCTs that could previously be assessed using the original threshold limits [10].

Study limitations

The main limitation of this study is the absence of a formal sample size calculation to determine the number of simulated RCTs needed to adequately capture the possible variations in baseline variables that may occur in real-life RCTs. However, owing to the novelty of its field of investigation, no past experience was available for the realistic estimation of marginal error, necessary for any meaningful calculation of sample size. Therefore, the sample size was arbitrarily set at N = 50 and multiplied by three variable domains (the minimum, maximum and median values of baseline variables that were extracted from 332 real-life RCTs). In hindsight, the chosen sample size, allowing the generation of 165,000 I^2^ data points for this study, appears to have been sufficiently large enough, to develop I^2^ threshold limits that allowed the testing of all 332 RCTs.

The study is further limited by the minimum/maximum ranges of baseline variables included in the 332 RCTs from a previous study. This trial cohort was originally identified from the reference list of 141 systematic review reports that in turn were randomly selected out of 423 systematic reviews, identified through a systematic literature search with January 24, 2024, as cut-off date [10]. While the RCTs spanned a wide range of medical specialities and were published between 1985 and 2023, making them broadly representative, any baseline data from trials outside this cohort will not have been included in the current study.

Due to these limitations, future selection bias testing of new RCTs may still generate I^2^ point estimates that fall outside the revised threshold limits, despite their improved utility. Future research could therefore focus on applying the selection bias test with the new I^2^ threshold limits on further RCTs, not included in this study and further revise the thresholds if necessary.

Notwithstanding its limitations, the results of this study support the utility of the trial-adjusted, SCT-based I^2^ test as method for the quantitative detection of selection bias and the assessment of the extent (%) of such bias within patient allocation to intervention groups in single RCTs. So far, clinical trial appraisal for selection bias is limited to qualitative text analysis, for example by using Cochrane’s RoB 2 as current gold standard in systematic reviews [15]. Such technique can only scan the text of trial reports for possible indicators of selection bias risk, but is unable to quantitatively analyze whether such bias actually exists. For this purpose, Berger and Exner pioneered a highly accurate statistical test for the detecting selection bias in RCTs. Their method is based on linear regression analysis conducted separately for each treatment group, with the reverse propensity score (the propensity of a patient being allocated to a specific intervention group) as the independent variable and the patient’s trial outcome as the dependent variable. However, this test requires individual patient level data, which is generally accessible only to trial authors and is rarely published as supplementary material of RCT reports [16].

In contrast, the trial-adjusted, SCT based I^2^ test can be applied by reviewers independently of the trial authors, using baseline data that are reported in most RCT publications.

## Conclusions

Within the limits of this study, the revised threshold limits increased the percentage of RCTs that could be tested for selection bias, using the trial-adjusted, SCT-based I^2^ test, from 71% to 100%. Rejection of both null hypotheses confirmed previous study results and indicates that the previously established statistically significant, positive relationship between selection bias levels (B%) and effect estimates (absolute RD) exists independently from the I^2^ threshold limit changes. Moreover, the findings of this study largely align previous test results and therefore do not invalidate the selection bias ratings of RCTs that could be assessed using the original I^2^ thresholds.

## Appendices

Appendix 1

All data is made fully available and downloadable without restriction at https://data.mendeley.com/datasets/629tr65jj2/1 (Sections 1-5).
